# A Small Physiological Electric Field Mediated Responses of Extravillous Trophoblasts Derived from HTR8/SVneo Cells: Involvement of Activation of Focal Adhesion Kinase Signaling

**DOI:** 10.1371/journal.pone.0092252

**Published:** 2014-03-18

**Authors:** Juan Zhang, Rongmei Ren, Xuefeng Luo, Ping Fan, Xinghui Liu, Shanshan Liang, Lei Ma, Ping Yu, Huai Bai

**Affiliations:** 1 Laboratory of Genetic Disease and Perinatal Medicine and Key Laboratory of Birth Defects and Related Diseases of Women and Children of Ministry of Education, West China Second University Hospital, Sichuan University, Chengdu, Sichuan, P. R. China; 2 Department of Obstetrics and Gynecology, West China Second University Hospital, Sichuan University, Chengdu, Sichuan, P. R. China; 3 Laboratory of Cell and Gene Therapy, West China Second University Hospital, Sichuan University, Chengdu, Sichuan, P. R. China; China Medical University, Taiwan

## Abstract

Moderate invasion of trophoblast cells into endometrium is essential for the placental development and normal pregnancy. Electric field (EF)-induced effects on cellular behaviors have been observed in many cell types. This study was to investigate the effect of physiological direct current EF (dc EF) on cellular responses such as elongation, orientation and motility of trophoblast cells. Immortalized first trimester extravillous trophoblast cells (HTR-8/SVneo) were exposed to the dc EF at physiological magnitude. Cell images were recorded and analyzed by image analyzer. Cell lysates were used to detect protein expression by Western blot. Cultured in the dc EFs the cells showed elongation, orientation and enhanced migration rate compared with non-EF stimulated cells at field strengths of 100 mV/mm to 200 mV/mm. EF exposure increased focal adhesion kinase (FAK) phosphorylation in a time-dependent manner and increased expression levels of MMP-2. Pharmacological inhibition of FAK impaired the EF-induced responses including motility and abrogated the elevation of MMP-2 expression. However, the expression levels of integrins like integrin α1, α5, αV and β1 were not affected by EF stimulation. Our results demonstrate the importance of FAK activation in migration/motility of trophobalst cells driven by EFs. In addition, it raises the feasibility of using applied EFs to promote placentation through effects on trophoblast cells.

## Introduction

Physiological electric fields (EFs) occur in embryonic development [1] and during wound healing [2], [3]. In vitro, a variety of cells respond to EFs with migration, elongation and orientation. These include epithelial cells, chondrocytes, bone cells, fibroblasts, smooth muscle cells and endothelial cells [4]–[8]. The mechanisms underlying these behaviors are unclear.

Formation of a functional placenta is essential for mammalian embryogenesis and fetal development. Extravillous cytotrophoblasts (EVTs) invade the underlying maternal tissue and then migrate into the wall of the uterine spiral arteries, which results in the remodeling of uterine vasculature. This process plays an important role in the mammalian placental development and is stringently regulated to ensure a successful pregnancy. Poor invasion of EVTs was believed to be associated with insufficient remodeling of the spiral arteries, which was typical pathological alterations in miscarriage [9], preeclampsia [10], and intrauterine growth restriction [11].

EVT invasion involves proteolytic degradation of decidual / endothelial extracellular matrix (ECM) in the direction of migration, then adhesion to ECM components, followed by active cell movement/migration through the degraded matrix [12]. For these processes, the action of proteases, particularly the matrix metalloproteinases (MMP-2 and MMP-9), is very important [13]. These proteases are secreted as latent enzymes, and their activities are further regulated by the local concentration of the major natural tissue inhibitors of metalloproteinases TIMP-1 and TIMP-2 [14]. Up to now the underlying mechanisms for the expression of matrix metalloproteinases were not fully understood.

Also known as protein tyrosine kinase 2 (PTK2), focal adhesion kinase (FAK) is a ubiquitously expressed non-receptor tyrosine kinase that functions as an important regulator of cell shape and adhesion in response to environmental signals [15], [16]. Clustering of integrins, the transmembrane receptors, can lead to the rapid recruitment of FAK to the focal adhesion complex and its concurrent phosphorylation on tyrosine [17], [18]. FAK-containing focal adhesions are thought to function as key sensory machineries that integrate extracellular signals, interconnect them with the cell's cytoskeleton and thus ultimately mediate complex cellular responses including cell motility and invasion [19].

The phosphorylation of tyrosine 397 in focal adhesion kinase (Tyr397), as in the case of integrin clustering by ECM, serves as the backbone of a scaffold that recruits additional intracellular signaling proteins to focal adhesions[20]. FAK has been implicated in the regulation of anchorage-dependent cell survival [21], [22] and in promoting cell motility [23], [24].

In the present study, we hypothesized that EF might regulate human EVT behaviors during early placentation. Owing to the restricted availability of primary human EVTs, studies were performed using immortalized first trimester EVT cell line, the HTR-8/SVneo cells. There are many reports available using this cell line as a model for EVT [25], [26]. We have determined the effects of EFs on HTR-8/SVneo cell motility as well as elongation and orientation, and characterized the cellular signaling molecule FAK involved. Furthermore, we also determined its effects on specific members of the MMP/TIMP system as well as expression of several integrin extracellular matrix receptors.

## Materials and Methods

### Reagents and Cell Culture

The HTR-8/SVneo trophoblast cell line was kindly provided by Dr. C.H. Graham, Queen's University, Kingston, Ontario, Canada [27]. The HTR-8/SVneo cells were maintained in RPMI-1640 medium supplemented with 10% fetal bovine serum (FBS),2 mM L-glutamine, penicillin(50 units/mL, and streptomycin(50 μg/ml) at 37°C in 5% CO_2_ [25]. Primary antibodies FAK, Phospho-FAK(Tyr397), MMP-2, MMP-9,TIMP-1, TIMP-2, integrins α1, α5, αV and β1 were purchased from Cell Signal technology Inc., BOSTER BIO-Engeneering, Ltd., or GeneTex, Inc. The secondary antibody horseradish peroxidase-conjugated IgG was the product of LKP, Inc. FAK inhibitor PF-573,228 and fluorescein isothiocyanate labeled Phalloidin were purchased from SIGMA. The thermo Scientific Pierce BCA Protein Assay Kit was used for determination of protein concentrations in cell lysates.

### Electrical stimulation

Human trophoblastic cells (HTR-8/SVneo) were cultured with RPMI-1640 (GIBCO) supplemented with 10% fetal bovine serum. The experimental setup and EF exposure protocols were similar to those reported previously [4] (Figure S1). In brief, the trophoblast cells at a density of ∼20×10^4^ cells/ml for morphological analysis or 5×10^5^ cells/ml for protein analysis were seeded in a specially made trough formed by two parallel (2 cm apart) strips of glass coverslip (No. 1, length of 22 mm or 50 mm) fixed to the base of the dish with silicone grease (Dow Corning, DC4).The cells were incubated for 24–48 hours (37°C, 5%CO2), allowing them to settle and adhere to the base of the dish, before a roof of No. 1 coverslip was applied and sealed with silicone grease. The final dimensions of the chamber, through which current was passed, were 22×10×0.2 mm or 50×10×0.2 mm. Agar-salt bridges not less than 15 cm long were used to connect silver/silver-chloride electrodes in beakers of Steinberg's solution (58 mM NaCl, 0.67 mM KCl, 0.44 mM Ca(NO3)2, 1.3 mM MgSO4, 4.6 mM Trizma base, pH 7.8–8.0), to pools of excess culture medium at either side of the chamber. This prevents diffusion of electrode products into the culture medium. EF strengths in the physiological range of 100, 150 and 200 mV/mm were used. Field strengths were measured directly at the beginning of, the end of and during each experiment. No fluctuations in field strength were observed. For drug inhibition experiments, the cells were incubated with the FAK inhibitor PF-573,228 (10 μM) for 1 hour before EF stimulation. The same concentration of drug was present during EF exposure in a CO2 incubator.

### Quantification of Cell Behavior

A series of images was taken with an image analyser immediately before field exposure and during 24 hours (1–5, 10 and 24 h) after field exposure [4], [7]. A minimum of ten fields per slide were taken, two slides were analyzed, and three to five replicates per period of time were evaluated. Individual frames were recorded and analyzed using light microscope Nikon Eclipse 80i and Image-Pro Plus software.

Mean migration rate was quantified over 5 h [4], [28], and elongation and orientation were quantified over 24 h.

#### Migration

The migration rate was defined as D/t, where D is the distance of a straight line connecting starting and end position of a cell over this period of time, and t is the duration of time.

#### Perpendicular alignment

Cell orientation was quantified as an orientation index (Oi) [4] (Figure S1). Oi = cos2(θ), where θ is the angle formed by the long axis of a cell with a line drawn perpendicular to the field lines. A cell with its long axis parallel to the vector of the EF will have an Oi of -1, and a cell with its long axis exactly perpendicular to the EF vector will have an Oi of 1. A randomly oriented population of cells will have an average Oi (defined by Σncos2θ/n) of 0. The significance of this two dimensional orientation distribution against randomness was calculated using Rayleigh's distribution [4].

#### Long: short axis ratio

A long:short axis ratio was calculated from the measurements made with the image analyser. This gives an objective assessment of elongation of the trophoblast cells in EFs.

Confocal microscopy to examine the organisation of filamentous actin (F-actin) in cells was as described previously [29]. In brief, EF-stimulated trophoblast cells or un-stimulated control cells were washed three times with PBS, fixed for 5 minutes in 3.7% formaldehyde, washed extensively in PBS, and then permeabilized with 0.1% Triton X-100. After three washes with PBS, cells were incubated with a 50 μg/ml fluorescent phalloidin conjugate solution in PBS for 40 minutes at room temperature, and then washed three times. Cells were immediately viewed using confocal laser scanning microscope.

### Western blot analyses

Trophoblast cells were stimulated by EF at different time-points. After each time point, the medium was aspirated and cells were lysed in 70 ul of lysis buffer (20 mM Tris-HCl, 10% glycerol, 0.2 mM EDTA, 0.137 M NaCl, 1% NP-40) supplemented with complete protease and phosphatase inhibitor cocktail (Roche). The cellular extract was incubated for 30 min on ice and then was subjected to centrifugation at 12000×g for 15 min at 4°C. The supernatant was collected, and the amount of protein in each sample was quantitated by BCA colorimetric assay using bovine serum albumin (BSA) as standard.

Samples containing 90 μg of total protein were loaded onto 10% SDS-polyacrylamide gels, and electrophoressed samples were transferred onto the polyvinylidene fluoride membrane as described before [30]. Individual blots were incubated at 4°C overnight with 1∶500/1000 dilution of rabbit polyclonal antibodies against FAK, phospho-FAK (Tyr397), MMP-2, MMP-9,TIMP-1, TIMP-2 (Cell Signaling Technology, Inc.), integrins α1, α5 (BOSTER BIO-Engeneering Ltd.) and αV, β1 (GeneTex, Inc.) followed by incubation with 1∶1000 dilution of HRP conjugated goat anti-rabbit IgG antibody (KPL, Inc.). Intensity of bands on Western blots were quantified using Quantity One software (Bio-Rad).

### Statistical analysis

The data were analyzed with the SPSS16.0 (SPSS Inc., Chicago, IL). For morphometric analysis, 60–90 cells were measured in each of 6–8 images from 3–5 separate experiments. For protein expression assessments, 3 separate experiments were taken. Means were compared using one-way analysis of variance (ANOVA) in group comparison. Two-tailed Student's t-test for unpaired data was applied as appropriate. A value of P<0.05 was considered statistically significant.

## Results

### Effects of EFs on trophoblast cell migration/motility

**Figure 1:**
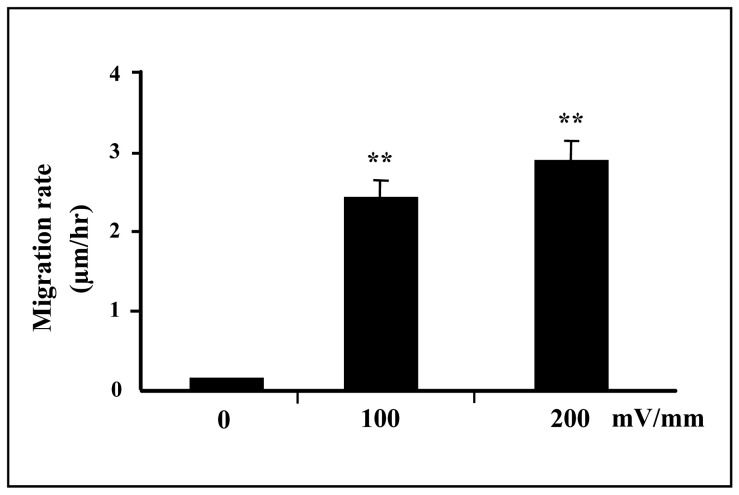
Motility enhancement of trophoblast cells in a small physiological EF. Trophoblast cells in culture exposed to an EF of 150/mm (over 5 hours) showed enhanced migration rate compared with non-EF stimulated cells (0 mV) at field strengths of 100 mV/mm and 200 mV/mm (**p<0.01, respectively).

Trophoblast cells exposed to small, applied EFs (150 mV/mm) showed motility enhancement (Supplemental Vedio-1). Cell migration rate was quantified as previously [4], [28]. Trophoblast cells exposed to dc EFs showed enhanced migration rate compared with non-EF stimulated cells at field strengths of 0 mV/mm (control), 100 mV/mm and 200 mV/mm [(0.12±0.02) μm/h, (2.47±0.25) μm/h and (2.90±0.28) μm/h, respectively] (Fig. 1).

### Effects of EFs on trophoblast cell elongation and alignment

**Figure 2:**
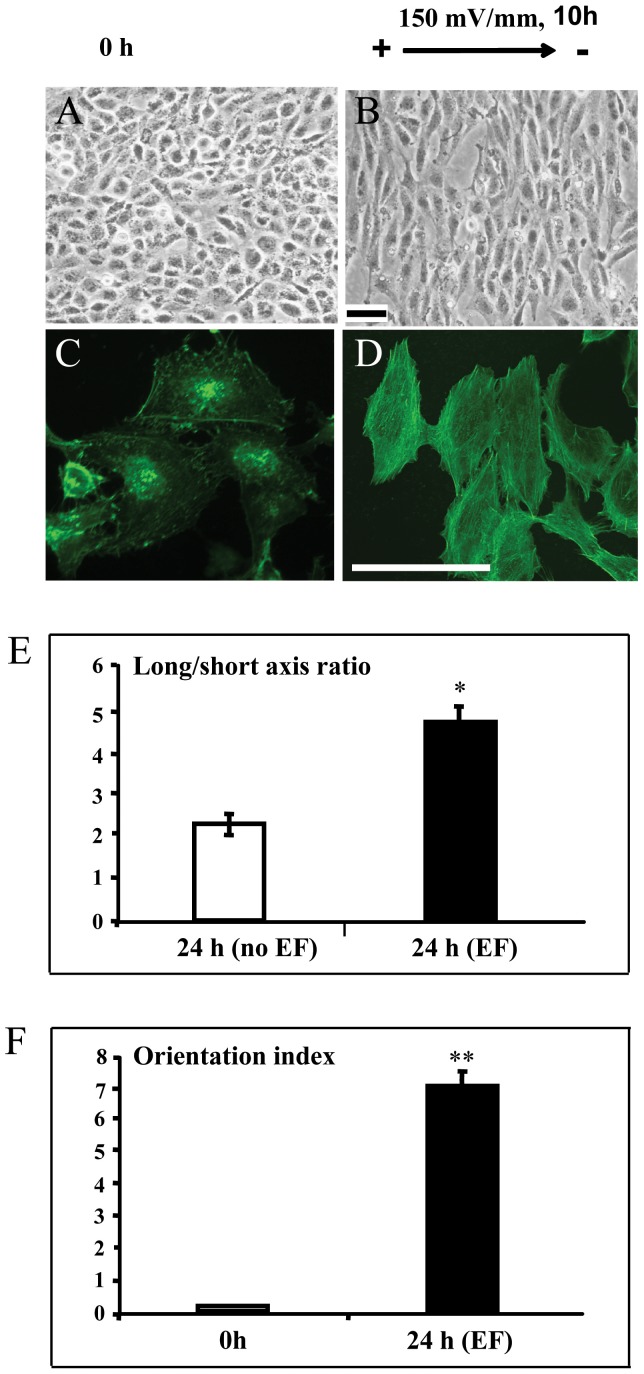
Trophoblast cells elongated and orientated perpendicularly in the electric field. Trophoblast cells exposed to small, applied EF (150 mV/mm) showed elongation and perpendicular orientation (Fig 2B), while control cells that were not subjected to EF showed no such responsiveness (Fig 2A). The morphology of control and EF treated cells stained by fluorescein isothiocyanate labeled Phalloidin (for staining F actin) showed in Fig 2C and D, respectively. Enhanced elongation and orientation compared with non-EF stimulated cells (control) at field strength of 150 mV/mm (Fig 2E and F, respectively). The error bars represent the S.E.*p<0.05 and **p<0.0001. Bar = 50 μm

Trophoblast cells elongated dramatically in an EF (Fig. 2B). By contrast, cells cultured with no EF showed no such response (Fig. 2A). We quantified the elongation of the cells using a long:short axis ratio (Materials and Methods). A perfectly round cell has a long:short axis ratio of 1. As cells elongate the ratio increases. Control cells (no EF) showed no increase in long:short axis over 24 h in culture (Fig. 2E).

Trophoblast cells cultured in dc EFs underwent a striking reorientation, with their long axis coming to lie perpendicular to the vector of the applied EF (Fig. 2B). Cells cultured without exposure to the EF had a typical morphology with random orientation of the long axis of cell body (Fig. 2A). The morphology of control and EF treated cells stained by fluorescein isothiocyanate labeled Phalloidin (for staining F actin), with the feature of the F actin alignment within the EF-treated cells, showed in Fig. 2C and D, respectively.

We quantified cell alignment using an orientation index (Materials and Methods): Oi. In cells oriented perpendicular to the field vector, the Oi = 1; cells parallel to the field vector, give an Oi of –1 and random orientation gives an Oi of 0. EF stimulated cells showed a significantly higher Oi (0.72±0.04, n = 305) at 24 hours of EF exposure, compared to cells without EF stimulation (0.05±0.03, n = 328)(P<0.0001) (Fig. 2F).

### Effects of EFs on activation of FAK

**Figure 3:**
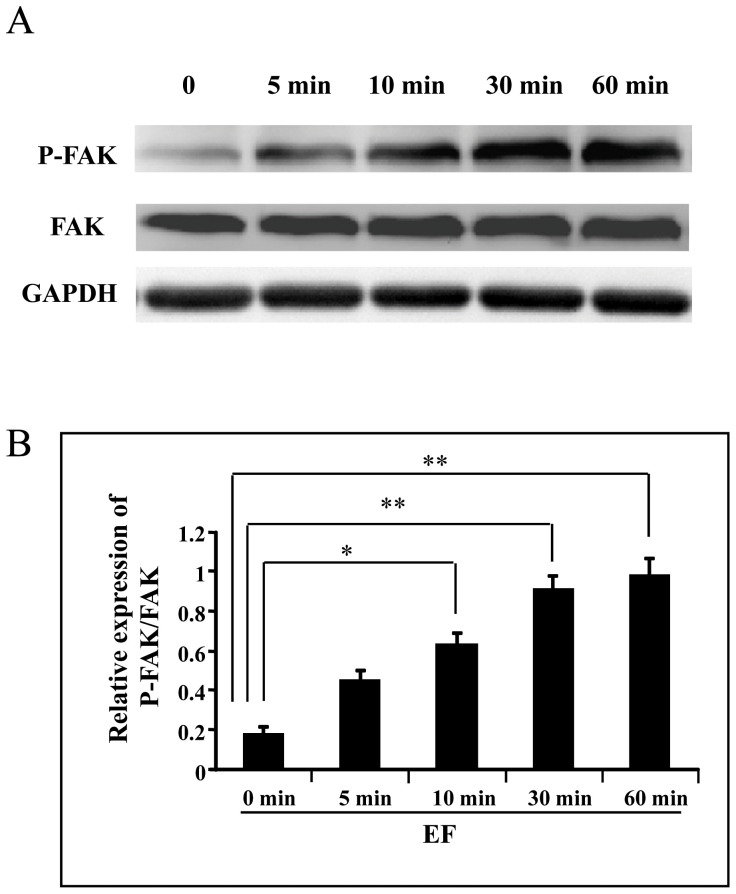
A small EF induces activation of FAK in trophoblast cells. A, FAK protein expression; B, quantification of FAK protein level. Trophoblast cells were stimulated by an EF of 150 mV/mm for 0, 5, 10, 30 and 60 min, respectively. Cell lysates were prepared after treatment of the cells with EF and Western blot was done for the FAK expression and activation as mentioned in Materials and Methods. FAK protein phosphorylations were increased significantly at the time points 10 to 60 min (compared with 0 h control), whereas FAK total protein levels showed no significant changes. The error bars represent the S.E. (*p<0.05, and **p<0.01).

FAK has been implicated as a signaling molecule involved in the early response of cell to stimulus. In this study trophoblast cells were treated with EF (150 mV/mm) for varying time periods (5, 10, 30 and 60 min) and cell lysates collected at specific time points were subjected for Western blot. There was a significantly higher levels of FAK (Tyr397) in trophoblast cells as compared to untreated control cells (Fig. 3 A and B) (p<0.05 or 0.01).

### Effect of EF on expression of MMPs and TIMPs

**Figure 4:**
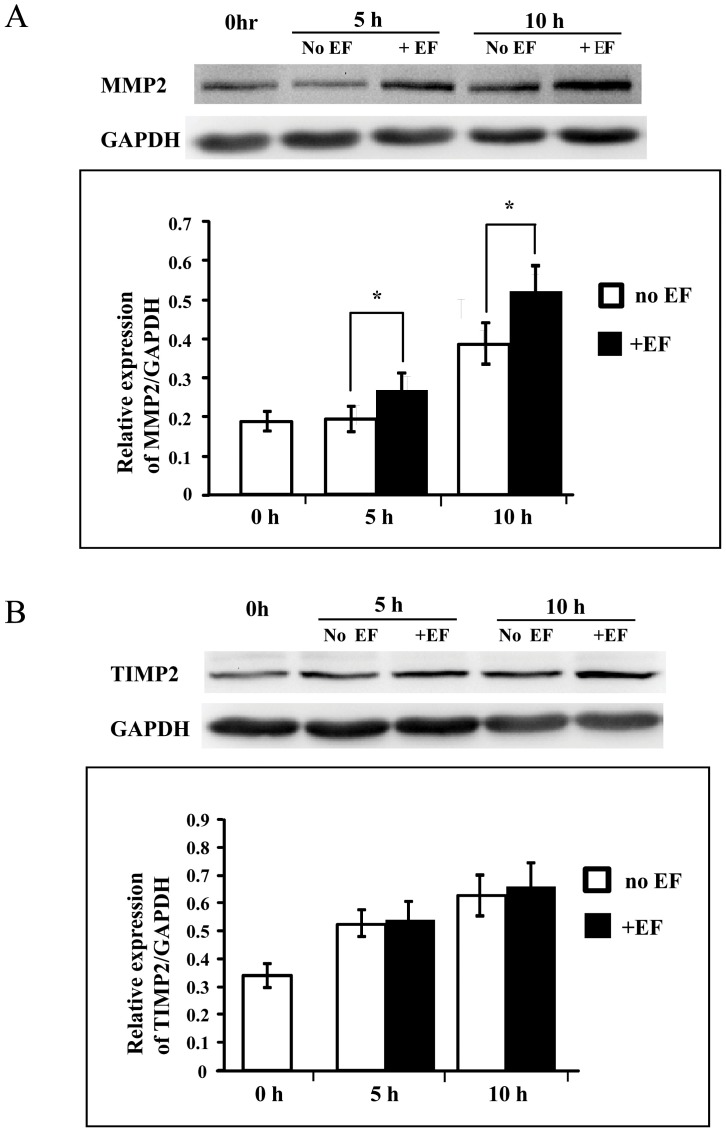
Effect of EF on the expression of MMP-2 and TIMP-2. EF stimulation enhanced the expression of MMP-2 (A), but not its endogenous inhibiting molecule TIMP-2 (B). Cell lysates were prepared after treatment of HTR-8/SVneo cells with EFs (150 mV/mm) for 5 and 10 h, respectively, and Western blot was done for the expression of MMP-2 and TIMP-2 as mentioned in Materials and Methods. A and B are representative of MMP-2 and TIMP-2 protein signal respectively, and house-keeping gene GAPDH as internal controls. Histograms depicting respective MMP-2 and TIMP-2 protein levels normalized to GAPDH. The error bars represent the S.E. (*p<0.05).

Expression of MMP-2, -9 and TIMP-1, -2 was analysed in trophoblast cells by Western blot after 5 and 10 h of EF treatment. There was a significant increase (p<0.05) in the expression of MMP-2 upon EF treatment (Fig. 4 A) while, EF treatment brought no significant changes in expression of TIMP-2, the endogenous molecule displaying specific inhibitory role against MMP-2 (Fig. 4 B). There were no significant changes in the expression of MMP-9 and its endogenous inhibitory molecule TIMP-1 in EF treated cells (not shown).

### Effect of EF on expression of integrins

**Figure 5:**
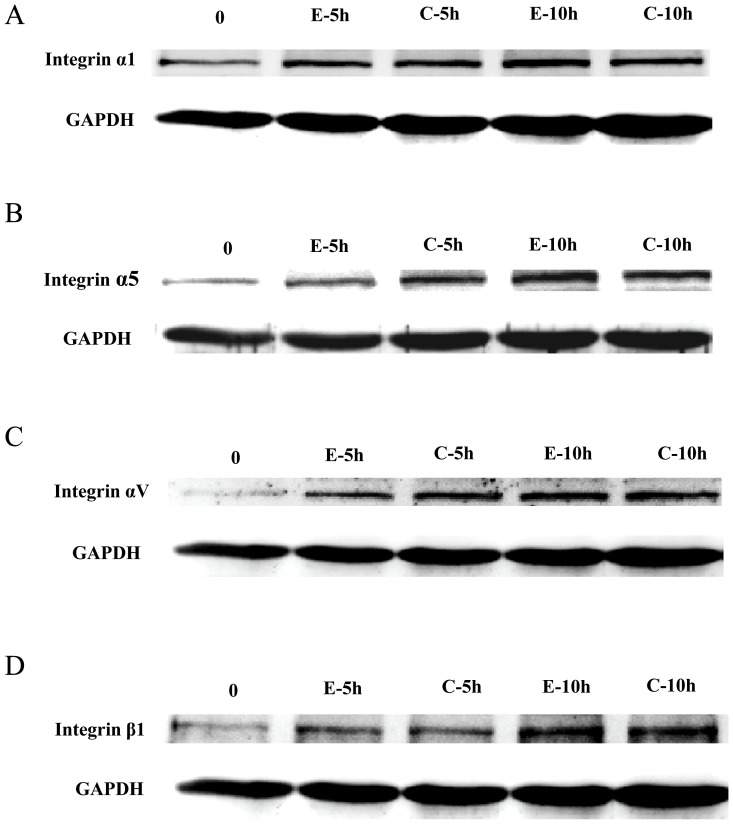
Effect of EF on expression of integrins. EF stimulation did not alter the expression of integrins α1, α5, αV and β1 in trophoblast cells (A, B, C and D, respectively). Cell lysates were prepared after treatment of HTR-8/SVneo cells with EFs (150 mV/mm) for 5 and 10 h, respectively, and Western blot was done for the expression of α1, α5, αV and β1 as mentioned in Materials and Methods.

Switching in the expression of integrins like integrin α1, α5 and β1 have been observed during the invasive differentiation of trophoblast cells [31], and integrin αV has been demonstrated to be involved in initial phase of metastasis in human melanoma cells [32]. Following EF treatment for 5–10 h no significant change in the expression of integrin α1, α5, αV and β1 were observed in trophoblast cells as compared to respective controls (Fig. 5).

### Inhibiting FAK activation abrogates the EFs mediated migration/motility and MMP-2 expression

**Figure 6:**
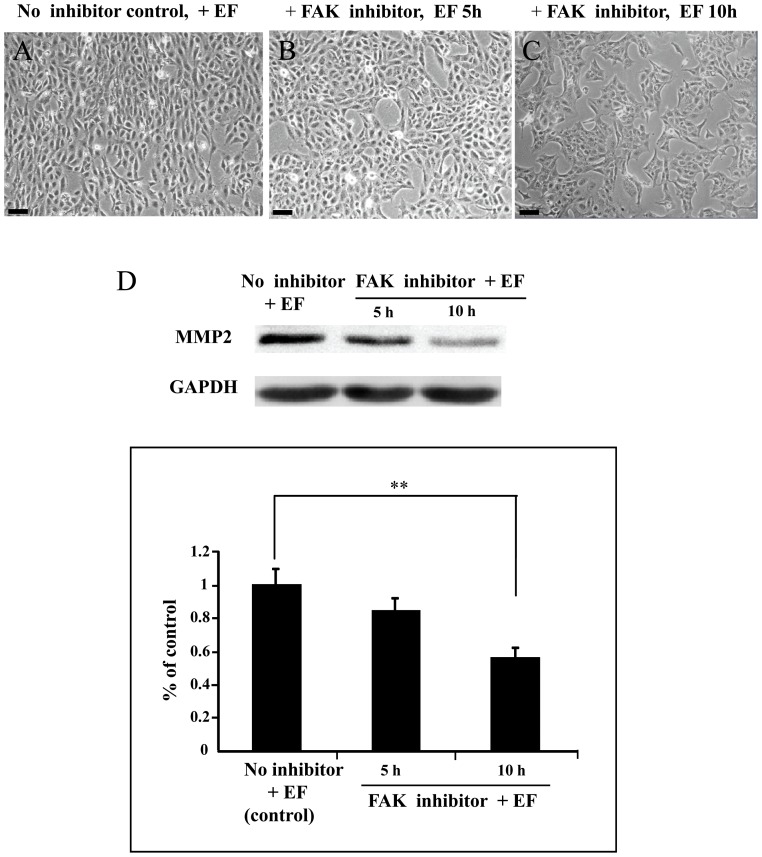
Inhibiting of FAK activation abrogates the EFs mediated cellular responses including migration/motility and MMP-2 expression. A, EF stimulated cells without FAK inhibitor treatment; B and C, EF stimulated cells pretreated with 10 μM FAK inhibitor for 5 h and 10 h,respectively. D, Trophoblast cells were pretreated with FAK inhibitor (10 μM) for 1 hour, and then the cells were stimulated by an EF of 150 mV/mm for 5 h and 10 h, respectively. Cells cultured in EF that had not been subjected to inhibitor treatment was used as the control. Cell lysates were prepared after treatment of the cells with EFs and Western blot was done for the expression of MMP-2. Histogram depicting MMP-2 protein levels normalized to GAPDH. The error bars represent the S.E. (**p<0.01). Bar = 50 μm.

To determine the significance of the FAK activation in EF mediated increase in migratory capacity and MMP-2 expression of trophoblast cells, the specific FAK inhibitor (PF-573,228) was used. Upon EF treatment at 150 mV/mm, there was an obvious responses of the PF-573,228-untreated cells (Fig. 6 A). However, cells treated by the FAK inhibitor led to a significant inhibition of cell responses including cell motility (Fig. 6 B and C). In addition, inhibiting FAK activation abrogates the EFs mediated increase in MMP-2 expression in trophoblast cells (Fig. 6 D).

## Discussion

In the present study we provided an evidence that EF could induce elongation,perpendicular orientation and motility of human trophoblast cells, which extended our knowledge concerning the EF –responsive cell types [33]. In addition, we demonstrated that EF could stimulate expression of MMP-2, and activate FAK, whereas inhibiting FAK activation abrogates the EFs mediated cellular responses including migration/motility and MMP-2 expression.

The experimentally measured electric fields near the cuts are 40 mV/mm and 100–200 mV/mm in bovine cornea and guinea-pig skin, respectively [2], [34]. The field strength will decrease exponentially within the extracellular space back from the wound but, at the edge, is 200 mV/mm. The field strength range of 100–200 mV/mm used in this study can be regarded as physiological.

Among the EFs induced cellular responses of trophoblast cells, elongation and orientation are obvious. The trophoblast cells demonstrated alignment in direction perpendicular to the applied electric field within 10 hours of the onset of stimulation, consistent with report of others applying similar culture conditions to fibroblast cells, smooth muscle cells as well as micro-and macro-vascular endothelial cells [8]. Interestingly, our cell elongation and alignment results match those of other studies with those type of cells, in which alignment perpendicular to the EF was obvious within 24 hours of stimulation [8], as opposed to 10 hours in the current experiment. Finkelstein et al reported that large cell sheets in DC fields responded with more sensitivity than single cells [35], perhaps because of a small voltage drop across the width of each individual cell as compared to a relatively large voltage drop across the width of cell sheets. A theory suggests that cells minimize the forces (or stress gradient) acting on them by aligning their long axis perpendicular to the principal direction of stretch. Similarly, the cell elongation and perpendicular alignment observed in the current study may be indicative of a mechanism to minimize the electric field gradient across the cell [36].

In addition, small dc EFs stimulate the motility of many cell types in humans and other mammalian species [33]. Studies demonstrated that small dc EFs are an overriding guidance cue that directs corneal epithelial cell migration in wound healing. In endothelial cells, small dc EFs induce cell migration, cell alignment, cell elongation and production of VEGF and IL-8 [7], [37], and therefore may provide a new approach to modulate angiogenesis. Our findings for the first time demonstrated that EFs could enhance trophoblast cell migration/motility (as well as elongation and orientation), suggesting that there might be a potential implication of EF on the effect of trophoblast cells relating to early implantation at the maternal-fetal interface. Tentatively, we propose that, in vivo trophoblast migration could be significantly enhanced with EF stimulation, and therefore EF has a therapeutic potential to the abnormal trophoblast invasion related to some complications of pregnancy, like miscarriage [9], preeclampsia [10], and intrauterine growth restriction [11]. The mechanism of transduction of electrical signal to the cells is likely via membrane proteins such as ion channels, growth factor receptors, and protein kinases [29], [38], [39].

Trophoblastic cells have been shown to express several proteases and their inhibitors but, the final outcome in terms of invasive behavior is regulated by the fine balance between the activating and inhibiting molecules. A number of cytokines and growth factors have been shown to increase the invasiveness of trophoblastic cells through changes in the expression of MMPs and TIMPs [40]–[42]. We observed that EF mediated increase in the expression of MMP-2 after 5 to 10 h treatment of HTR-8/SVneo cells, and no effects of EF were seen on TIMP-2 (as well as MMP-9 and TIMP-1) expression in the cells, which were reflected by Western blot analysis.

This observation indicates that EF might be served as a novel cue involved in mediating invasion of trophoblast cells. Beyond MMPs and TIMPs, adhesion molecules like integrins also play an important role in invasion of trophoblastic cells [31]. Treatment of HTR-8/SVneo cells with EF did not show any significant change in the expression of integrins α1, α5, αV and β1, suggesting that these molecules might not play an important role in EF-mediated invasion of trophoblastic cells.

A role for FAK in promoting cell motility has been established by results using genetic approaches [23]. FAK-deficient embryos were able to implant, but were defects in the development of the mesoderm, within which consisted of cells with the highest migratory ability. In addition, in vitro study demonstrated that cells from both the mesoderm and the endoderm exhibited impaired migratory ability. In contrast, FAK overexpression displayed an enhanced migratory ability of fibroblasts[43] and keratinocytes[44], and has been consistently observed in highly metastatic melanoma cell lines and in invasive and metastatic breast and colon tumors[45].

Besides EF increased migration rate and phosphorylation of focal adhesion kinase, it also had effect on cell cytoskeleton rearrangement. This provided a potential mechanism for the observed induction of trophoblast motility. Indeed, in MCF7 breast cancer cell line, motility and invasiveness of the cells under corticotropin-releasing factor stimulation showed involvement of FAK phosphorylation and actin filament reorganization[46].

Recently, Ruan and co-workers made an exciting discovery that activation of the epithelial Na^+^ channel could trigger prostaglandin E2 release and production required for embryo implantation[47]. Indeed, all cell types and intracellular organelles maintain transmembrane electrical potentials owing to asymmetric ion transport. For instance, in mammalian corneal epithelium naturally occurring EFs in tissue arise from polarized ion transport: i.e. the enriched Na^+^ channels (and Cl^-^ transporter) in apical domain of the cell [48]. In human adipose tissue derived stem cells there are channels for a Ca^2+^-activated k^+^ current, a transient outward k^+^ current, a delayed rectifier-like k^+^ current, and also a tetrodotoxin-sensitive transient inward Na^+^ current[49].It has been suggested that in almost all system crucial cellular behaviours, such as division, migration and differentiation, take place within an excellular microenvironment in witch standing voltage gradients persist for several hours or even for a few days [50], [51]. Based on the above reports and the present finding that trophoblast cells are responsive to EF signal, we speculate that EF might exert a profound influence on the trophoblast cell function via direct activation of ion channels. An interesting area for future work might be to investigate whether ion channels, including the epithelial Na^+^ channel on the trophoblast cells, are involved in the transduction of electrical stimulus and thus establish a potential significance in EF-mediated cellular functions linking to embryogenesis and fetal development.

In conclusion, EF stimulation induces trophoblast cell responses such as elongation, orientation and motility as well as cytoskeleton rearrangement, and upregulates the expression of MMP-2 in the cells. Such an upregulation is likely a specific effect, at least for the time points tested. Activation of FAK is required for the cellular responses and upregulation of MMP-2. Small dc EF-induced trophoblast migration/motility may be a promising source of potential future therapeutic strategies, although further studies need to be validated using primary isolates of EVT cells.

## Supporting Information

Figure S1 **Schematic diagram shows experimental design of culture chamber and field application, and the method of quantification of cell orientation.** (A) chamber constructed within a tissue culture plastic dish, viewed from above. (B) side-on view includes dc power supply and Ag /AgCl electrodes isolated from the culture chamber using agar-gelled salt bridges. (C) Measurement of electric field using an electric meter. (D) Perpendicular alignment of cells to electric vector would give an orientation index (Oi) approaching 1, parallel alignment of cells to electric vector an Oi approaching –1, while random orientated cells an Oi of 0.
(TIF)

Video S1 **A real-time imaging of live cells cultured in the electric field.** EF stimulation enhanced motility of the trophoblast cells in culture exposed to an EF of 150 mV/mm (over 5 hours).
(AVI)

## References

[1] Robinson KR, Messerli MA (1996) Electric embryos: the embryonic epithelium as a generator of developmental information. In: McCaig CD, editor.Nerve Growth and Nerve Guidance.\London, UK: Portland Press. pp.131–150.

[2] Chiang M, Robinson KR, Vanable JW (1992) Electrical fields in the vicinity of epithelial wounds in the isolated bovine eye. Exp Eye Res 54: 999–1003. 1521590 10.1016/0014-4835(92)90164-n

[3] Sta Iglesia DD, Vanable JW (1998) Endogenous lateral electric fields around bovine corneal lesions are necessary for and can enhance normal rates of wound healing. Wound Repair Reg 6: 531–542. 10.1046/j.1524-475x.1998.60606.x 9893173

[4] Zhao M, Agius-Fernandez A, Forrester JV, McCraig CD (1996) Orientation and directed migration of cultured corneal epithelial cells in small electric fields are serum dependent. J Cell Sci 109: 1405–1414. 8799828 10.1242/jcs.109.6.1405

[5] Chao P-HG, Roy R, Mauck RL, Liu W, Valhmu WB, et al (2000) Chondrocyte Translocation Response to Direct Current Electric Fields. Journal of Biomechanical Engineering 122: 261–267. 10923294 10.1115/1.429661

[6] Ferrier J, Ross SM, Kanehisa J, Aubin JE (1986) Osteoclasts and osteoblasts migrate in opposite directions in response to a constant electrical field. J Cell Physiol 129: 283–288. 3782308 10.1002/jcp.1041290303

[7] Zhao M, Bai H, Forrester JV, McCaig CD (2004) Electrical stimulation directly induces pre-angiogenic responses in vascular endothelial cells by signaling through VEGF receptors. J Cell Sci 117: 397–405. 14679307 10.1242/jcs.00868 PMC1459284

[8] Bai H, McCaig CD, Forrester JV, Zhao M (2004) Direct Current Electrical Fields Induce Distinct Pre- Angiogenic Responses in Micro- and Macro-vascular Cells. Arterioscler Thromb Vasc Biol 24: 1234–1239. 15130919 10.1161/01.ATV.0000131265.76828.8a

[9] Ball E, Bulmer JN, Ayis S, Lyall F, Robson SC (2006) Late sporadic miscarriage is associated with abnormalities in spiral artery transformation and trophoblast invasion. J Pathol 208: 535–542. 16402350 10.1002/path.1927

[10] Kadyrov M, Schmitz C, Black S, Kaufmann P, Huppertz B (2003) Pre-eclampsia and maternal anaemia display reduced apoptosis and opposite invasive phenotypes of extravillous trophoblast. Placenta 24: 540–548. 12744931 10.1053/plac.2002.0946

[11] Khong TY, De Wolf F, Robertson WB, Brosens I (1986) Inadequate maternal vascular response to placentation in pregnancies complicated by pre-eclampsia and by small-for-gestational age infants. Br J Obstet Gynaecol 93: 1049–1059. 3790464 10.1111/j.1471-0528.1986.tb07830.x

[12] Mareel M, Leroy A (2003) Clinical, cellular, and molecular aspects of cancer invasion. Physiological Reviews 83: 337–376. 12663862 10.1152/physrev.00024.2002

[13] Staun-Ram E, Goldman S, Gabarin D, Shalev E (2004) Expression and importance of matrix metalloproteinase 2 and 9 (MMP-2 and -9) in human trophoblast invasion. Reproductive Biology and Endocrinology 2: 59. 15294019 10.1186/1477-7827-2-59 PMC516041

[14] Huppertz B, Kertschanska S, Demir AY, Frank HG, Kaufmann P (1998) Immunohistochemistry of matrix metalloproteinases (MMP), their substrates, and their inhibitors (TIMP) during trophoblast invasion in the human placenta. Cell and Tissue Research 291: 133–148. 9394051 10.1007/s004410050987

[15] Mitra SK, Hanson DA, Schlaepfer DD (2005) Focal adhesion kinase: in command and control of cell motility. Nat Rev Mol Cell Biol 6: 56–68. 15688067 10.1038/nrm1549

[16] Schaller MD (2010) Cellular functions of FAK kinases: insight into molecular mechanisms and novel functions. J Cell Sci 123: 1007–1013. 20332118 10.1242/jcs.045112

[17] Berrier AL, Yamada KM (2007) Cell-matrix adhesion. J Cell Physiol 213: 565–273. 17680633 10.1002/jcp.21237

[18] Geiger B, Spatz JP, Bershadsky AD (2009) Environmental sensing through focal adhesions. Nat Rev Mol Cell Biol 10: 21–33. 19197329 10.1038/nrm2593

[19] Cary LA, Guan JL (1999) Focal adhesion kinase in integrin-mediated signaling. Front Biosci 4: D102–113. 9889179 10.2741/cary

[20] Schlaepfer DD, Hauck CR, Sieg DJ (1999) Signaling through focal adhesion kinase. Prog Biophys Mol Biol 71: 435–478. 10354709 10.1016/s0079-6107(98)00052-2

[21] Frisch SM, Vuori K, Ruoslahti E, Chan-Hui PY (1996) Control of adhesion-dependent cell survival by focal adhesion kinase. J Cell Biol 134: 793–799. 8707856 10.1083/jcb.134.3.793 PMC2120934

[22] Almeida EA, Ilić D, Han Q, Hauck CR, Jin F, et al (2000) Matrix survival signaling: from fibronectin via focal adhesion kinase to c-Jun NH(2)-terminal kinase. J Cell Biol 149: 741–754. 10791986 10.1083/jcb.149.3.741 PMC2174844

[23] Ilić D, Furuta Y, Kanazawa S, Takeda N, Sobue K, et al (1995) Reduced cell motility and enhanced focal adhesion contact formation in cells from FAK-deficient mice. Nature 377: 539–544. 7566154 10.1038/377539a0

[24] Fox GL, Rebay I, Hynes RO (1999) Expression of DFak56, a Drosophila homolog of vertebrate focal adhesion kinase, supports a role in cell migration in vivo. Proc Natl Acad Sci USA 96: 14978–14983. 10611323 10.1073/pnas.96.26.14978 PMC24758

[25] Benaitreau D, Dos Santos E, Leneveu MC, Alfaidy N, Feige JJ, et al (2010) Effects of adiponectin on human trophoblast invasion. J Endocrinol 207: 45–53. 20675305 10.1677/JOE-10-0170

[26] Johnsen GM, Basak S, Weedon-Fekjaer MS, Staff AC, Duttaroy AK (2011) Docosahexaenoic acid stimulates tube formation in first trimester trophoblast cells, HTR8/SVneo. Placenta 32: 626–632. 21741084 10.1016/j.placenta.2011.06.009

[27] Graham CH, Hawley TS, Hawley RG, MacDougall JR, Kerbel RS, et al (1993) Establishment and characterization of first trimester human trophoblast cells with extended lifespan. Exp Cell Res 206: 204–211. 7684692 10.1006/excr.1993.1139

[28] Erickson CA, Nuccitelli R (1984) Embryonic fibroblast motility and orientation can be influenced by physiological electric fields. J Cell Biol 98: 296–307. 6707093 10.1083/jcb.98.1.296 PMC2112998

[29] Zhao M, Dick A, Forrester JV, McCaig CD (1999) Electric field directed cell motility involves up-regulated expression and asymmetric redistribution of the epidermal growth factor receptors and is enhanced by fibronectin and by laminin. Mol Biol Cell 10: 1259–1276. 10198071 10.1091/mbc.10.4.1259 PMC25266

[30] Chang MM, Lovett J (2011) A laboratory exercise illustrating the sensitivity and specificity of Western blot analysis.Biochem Mol Biol Educ. 39: 291–297. 10.1002/bmb.20501 21774057

[31] Damsky CH, Librach C, Lim KH, Fitzgerald ML, McMaster MT, et al (1994) Integrin switching regulates normal trophoblast invasion. Development 120: 3657–3666. 7529679 10.1242/dev.120.12.3657

[32] Felding-Habermann B, Mueller BM, Romerdahl CA, Cheresh DA (1992) Involvement of integrin alpha V gene expression in human melanoma tumorigenicity. J Clin Invest 89: 2018–2022. 1376331 10.1172/JCI115811 PMC295910

[33] McCaig CD, Zhao M (1997) Physiological electrical fields modify cell behaviour. Bioessays 19: 819–826. 9297973 10.1002/bies.950190912

[34] Barker AT, Jaffe LF, Vanable JW Jr (1982) The glabrous epidermis of cavies contains a powerful battery. Am J Physiol 242: R358–R366. 7065232 10.1152/ajpregu.1982.242.3.R358

[35] Finkelstein E, Chang W, Chao PH, Gruber D, Minden A, et al (2004) Roles of microtubules, cell polarity and adhesion in electric-field-mediated motility of 3T3 fibroblasts. J Cell Sci 117: 1533–1545. 15020680 10.1242/jcs.00986

[36] Nishimura KY, Isseroff RR, Nuccitelli R (1996) Human keratinocytes migrate to the negative pole in direct current electric fields comparable to those measured in mammalian wounds.J Cell Sci. 109: 199–207. 10.1242/jcs.109.1.199 8834804

[37] Bai H, Forrester JV, Zhao M (2011) DC electric stimulation upregulates angiogenic factors in endothelial cells through activation of VEGF receptors. Cytokine 55: 110–115. 21524919 10.1016/j.cyto.2011.03.003 PMC4437767

[38] Djamgoz MBA, Mycielska M, Madeja Z, Fraser SP, Korohoda W (2001) Directional movement of rat prostate cancer cells in direct-current electric field: involvement of voltagegated Na+ channel activity. J Cell Sci 114: 2697–2705. 11683396 10.1242/jcs.114.14.2697

[39] Meng X, Arocena M, Penninger J, Gage FH, Zhao M, et al (2011) PI3K mediated electrotaxis of embryonic and adult neural progenitor cells in the presence of growth factors. Exp Neurol 227: 210–217. 21092738 10.1016/j.expneurol.2010.11.002 PMC3821524

[40] Qiu Q, Yang M, Tsang BK, Gruslin A (2004) EGF-induced trophoblast secretion of MMP-9 and TIMP-1 involves activation of both PI3K and MAPK signalling pathways. Reproduction 128: 355–63. 15333786 10.1530/rep.1.00234

[41] Fitzgerald JS, Tsareva SA, Poehlmann TG, Berod L, Meissner A, et al (2005) Leukemia inhibitory factor triggers activation of signal transducer and activator of transcription 3, proliferation, invasiveness, and altered protease expression in choriocarcinoma cells. Int J Biochem Cell Biol 37: 2284–2296. 16125646 10.1016/j.biocel.2005.02.025

[42] Jovanovic M, Stefanoska I, Radojcic L, Vicovac L (2010) Interleukin-8 (CXCL8) stimulates trophoblast cell migration and invasion by increasing levels of matrix metalloproteinase (MMP)2 and MMP9 and integrins alpha5 and beta1. Reproduction 139: 789–798. 20133364 10.1530/REP-09-0341

[43] Cary LA, Chang JF, Guan J-L (1996) Stimulation of cell migration by overexpression of focal adhesion kinase and its association with Src and Fyn. J Cell Sci 109: 1787–1794. 8832401 10.1242/jcs.109.7.1787

[44] Gates RE, King Jr LE, Hanks SK, Nanney LB (1994) Potential role for focal adhesion kinase in migrating and proliferating keratinocytes near epidermal wounds and in culture. Cell Growth Differ 5: 891–899. 7986754

[45] Owens LV, Xu L, Craven RJ, Dent GA, Weiner TM, et al (1995) Overexpression of the focal adhesion kinase (p125FAK) in invasive human tumors. Cancer Res 55: 2752–2755. 7796399

[46] Androulidaki A, Dermitzaki E, Venihaki M, Karagianni E, Rassouli O, et al (2009) Corticotropin Releasing Factor promotes breast cancer cell motility and invasiveness. Mol Cancer 8: 30. 19490624 10.1186/1476-4598-8-30 PMC2697132

[47] Ruan YC, Guo JH, Liu X, Zhang R, Tsang LL, et al (2012) Activation of the epithelial Na+ channel triggers prostaglandin E2 release and production required for embryo implantation. Nat Med 18: 1112–1117. 22729284 10.1038/nm.2771

[48] McCaig CD, Song B, Rajnicek AM (2009) Electrical dimensions in cell science. J Cell Sci 122: 4267–4276. 19923270 10.1242/jcs.023564

[49] Bai X, Ma J, Pan Z, Song YH, Freyberg S, et al (2007) Electrophysiological properties of human adipose tissue-derived stem cells. Am J Physiol Cell Physiol 293: C1539–1550. 17687001 10.1152/ajpcell.00089.2007

[50] Levin M (2007) Large-scale biophysics: ion flows and regeneration. Trends Cell Biol 17: 261–270. 17498955 10.1016/j.tcb.2007.04.007

[51] McCaig CD, Rajnicek AM, Song B, Zhao M (2005) Controlling cell behavior electrically: current views and future potential. Physiol Rev 85: 943–978. 15987799 10.1152/physrev.00020.2004

