# Supplementary figures and images for: A Small Physiological Electric Field Mediated Responses of Extravillous Trophoblasts Derived from HTR8/SVneo Cells: Involvement of Activation of Focal Adhesion Kinase Signaling
Source: PLoS One. 2014 Mar 18;9(3):e92252. doi: 10.1371/journal.pone.0092252 (PMC3958492; doi:10.1371/journal.pone.0092252)

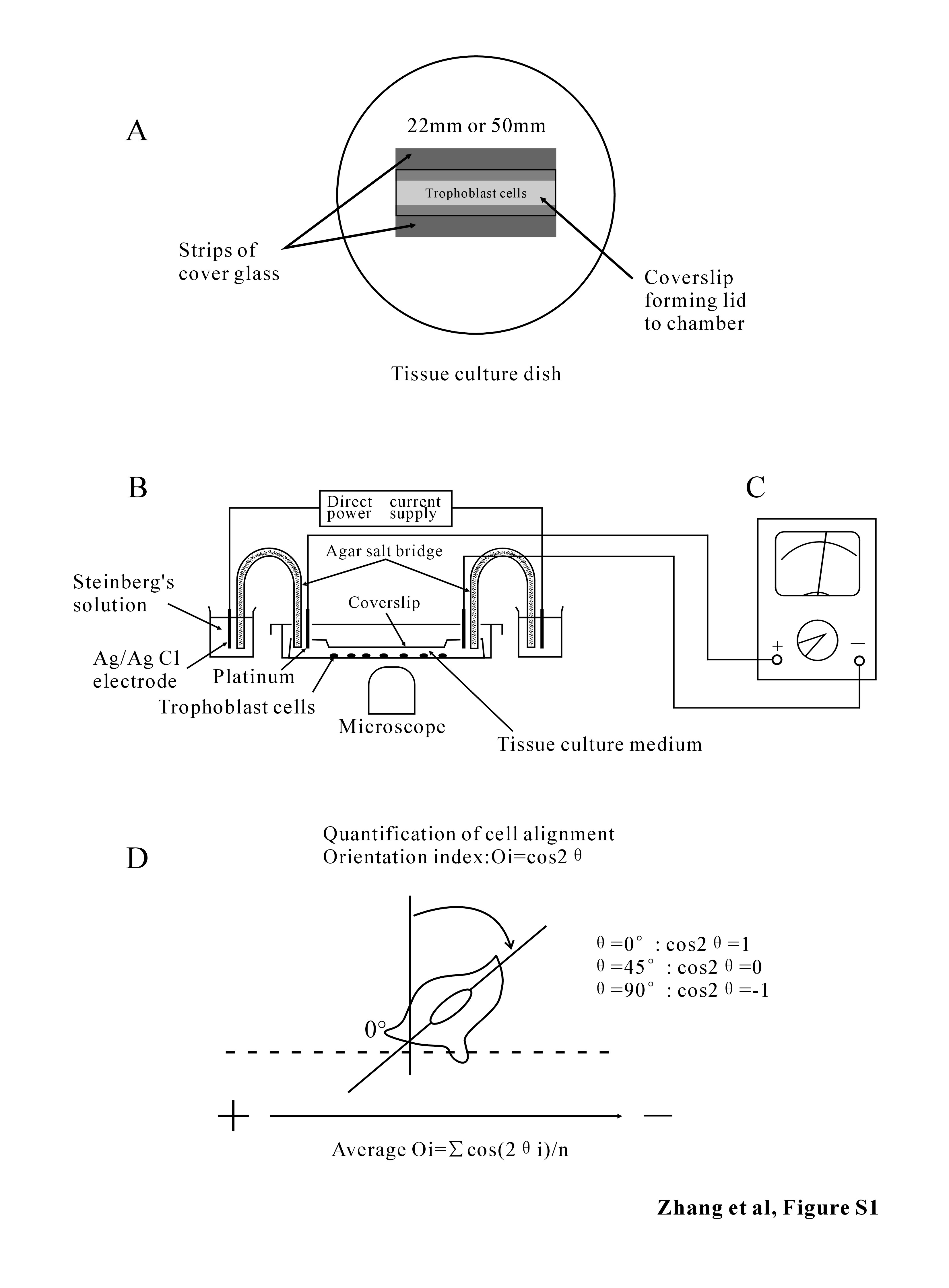

Supplement: Figure S1 — Schematic diagram shows experimental design of culture chamber and field application, and the method of quantification of cell orientation. (A) chamber constructed within a tissue culture plastic dish, viewed from above. (B) side-on view includes dc power supply and Ag /AgCl electrodes isolated from the culture chamber using agar-gelled salt bridges. (C) Measurement of electric field using an electric meter. (D) Perpendicular alignment of cells to electric vector would give an orientation index (Oi) approaching 1, parallel alignment of cells to electric vector an Oi approaching –1, while random orientated cells an Oi of 0. (TIF) [file pone.0092252.s001.tif]
